# DIFFERENTIAL EFFECTS OF VARYING DOSES OF DIETARY NITRATE ON MUSCLE FUNCTION AND BLOOD PRESSURE IN OLDER SUBJECTS

**DOI:** 10.1093/geroni/igz038.320

**Published:** 2019-11-08

**Authors:** Andrew R Coggan, Edgar Gallardo, Derrick A Gray, Richard Hoffman, Ranjani Moorthi

**Affiliations:** Indiana University Purdue University Indianapolis, Indianapolis, Indiana, United States

## Abstract

We have recently demonstrated that dietary nitrate, a source of nitric oxide via the enterosalivary pathway, can improve muscle contractile function in healthy older men and women. Nitrate ingestion has also been shown to reduce blood pressure in older individuals. However, the optimal dose for eliciting these beneficial effects is unknown. We therefore performed a randomized, double-blind, crossover study to determine the effects of ingesting 3.3 mL/kg of beetroot juice (BRJ) containing 0, 212, or 425 µmol/kg of nitrate in six healthy older (age 69±3 y) subjects. Maximal knee extensor speed (Vmax) and power (Pmax) were measured 2 h after BRJ ingestion using isokinetic dynamometry; blood pressure was monitored periodically throughout each study. Mean arterial pressure (in mmHg) was lower (P<0.05) after the high (80±4) vs. the low (84±3) or placebo (88±2) doses. Vmax (in rad/s), however, was higher (P<0.05) after the low dose (11.7±0.8), but not the high dose (10.8±1.0), compared to the placebo (10.5±1.0). Pmax (in W/kg) also tended to be higher (P=0.11) in the low (3.9±0.5) compared to the placebo (3.7±0.5) or high (3.7±0.5) trials. Five out of six subjects achieved a higher Vmax and Pmax after the low vs. the high dose. We conclude that dietary nitrate has differential effects on muscle function and blood pressure in older individuals. A high dose of nitrate intake further lowers blood pressure but does not enhance muscle contractility as much as a lower dose. Supported by Indiana University Purdue University Indianapolis and by the NIA (R21 AG053606)

